# *Editorial Commentary:* Single-Dose Primaquine as Gametocytocidal Treatment in Patients With Uncomplicated Falciparum Malaria

**DOI:** 10.1093/cid/cis962

**Published:** 2012-11-21

**Authors:** Arjen M. Dondorp

**Affiliations:** 1 Mahidol-Oxford Tropical Medicine Research Unit, Faculty of Tropical Medicine, Mahidol University, Bangkok, Thailand; 2 Centre for Tropical Medicine, Churchill Hospital, University of Oxford, United Kingdom

**(See the Major Article by Sutanto et al on pages 685–93** **and the Electronic Article by Das et al on pages e48–e58.)**

The world has seen a significant reduction in the burden of *Plasmodium falciparum* malaria over the last decade, which can to a large extent be attributed to the wide deployment of insecticide-treated bed nets and the introduction of the highly effective artemisinin combination therapies. Effective early asexual stage antimalarial treatment will prevent the transition to early developing gametocytes, but in addition the artemisinin derivatives affect young gametocytes, which augments their transmission-reducing capacity. However, mature gametocytes responsible for transmission of the disease can be present on first presentation and last for several weeks even with artemisinin-based combination therapy (ACT). The 8-aminoquinolone primaquine is the only drug in general use with gametocytocidal activity against these mature forms, and can be an important adjunct to reduce malaria transmission essential for the ultimate aim of malaria elimination. This aim has gained importance with the emergence of partial resistance to artemisinins on the Cambodian-Thai and Thai-Myanmar borders, which has the potential to spread to other places in the region [1]. Containment of the problem implies the elimination of falciparum malaria in areas of artemisinin resistance, since with continuing drug pressure the remaining parasite populations will be the most resistant [2]. Deployment of primaquine is important to reach this goal, and its use is recommended in many of the region's national treatment guidelines. However, in practice the drug is not widely used because of safety concerns, in particular the drug's oxidative capacity causing intravascular hemolysis in patients with glucose-6-phosphate dehydrogenase (G6PD) deficiency. Treatment with primaquine, which has no effect on the blood-stage parasites causing the disease, has no benefit for the individual patient, so that there will be low tolerance for drug-related adverse events.

In this context, the well-designed and carefully executed open randomized controlled trial by Sutanto and colleagues published in this issue of *Clinical Infectious Diseases* makes a contribution to establish the risk-benefit profile of primaquine as an adjunct to ACT in the treatment of uncomplicated falciparum malaria in South Sumatra, Western Indonesia [3]. Prevalence of G6PD deficiency is around 5% in North Sumatra [4], but no published data are available for South Sumatra. In the current study, patients were screened prior to randomization with a qualitative fluorescent spot test to detect G6PD deficiency. G6PD deficiency was an exclusion criterion for enrollment in the trial, but surprisingly all 374 patients who provided informed consent had normal test results. Patients were randomized to receive 3 days of treatment with dihydroartemisinin-piperaquine with or without the addition of a single dose of 0.75 mg/kg primaquine given on the third day of treatment. Primaquine had a large effect on gametocyte carriage: In the 21% of patients with microscopically detectable gametocytes on day 3, clearance was about twice as fast with primaquine (hazard ratio, 2.42 [95% confidence interval, 1.39–4.19], *P* = .002). In patients without gametocytes on day 3, none of the patients in either study arm developed microscopically detectable gametocytemia. The addition of primaquine was well tolerated in this population. In particular, gastrointestinal complaints were similar in the 2 treatment arms, as were the hemoglobin concentrations on 7 and 42 days after start of treatment. Follow-up was remarkably complete. The authors conclude that in settings where screening for G6PD is feasible, a single gametocytocidal dose of primaquine should be added to ACT for the treatment of falciparum malaria in low-transmission settings. However, as the authors recognize, routine screening for G6PD may not be feasible in most treatment settings.

More practical point-of-care dipstick format tests to detect G6PD deficiency are under development, which will facilitate testing in the future. Prevalence of G6PD deficiency in malaria-endemic areas is usually somewhere between 5% and 20% [5]. The G6PD gene is located on the X chromosome and >140 functional polymorphisms are known to confer reductions in G6PD enzyme activity. The more common ones are well characterized and differ importantly in their effect on enzyme activity. Thus, for safe deployment of primaquine in a malaria-endemic area, information is needed on the hemolytic effects of the proposed primaquine regimen in malaria patients with the common G6PD genotypes prevalent in that area. These studies are currently being planned in several Asian countries. However, there is an urgent need now for wider, yet safe, deployment of primaquine in the context of containment and elimination of artemisinin-resistant malaria.

For this reason, the World Health Organization (WHO) has recently convened an evidence review group on the use of single-dose primaquine as a gametocytocidal drug in the treatment of falciparum malaria [6]. On the basis of a review of all published and unpublished data, the group concluded that a single dose as low as 0.25 mg/kg might be sufficient to block transmission and can be deployed safely without prior testing for G6PD enzyme activity. The efficacy of this low dose is related to its inhibitory effect on transmissibility, as assessed from parasite oocyst and sporozoite numbers in the mosquito, which precedes the effect on gametocyte densities and is achieved at lower primaquine doses [7, 8]. As a result of this consultation, the WHO now recommends in areas threatened by artemisinin resistance, as well as in elimination areas (and where single-dose primaquine as a gametocytocide for falciparum malaria is not yet being implemented), a single 0.25 mg base/kg primaquine dose for all patients with parasitologically confirmed falciparum malaria on the first day of treatment in addition to ACT without prior testing for G6PD deficiency. Pregnant women and infants <1 year of age are excluded from this recommendation [9].

The timing of the primaquine dose in the revised guideline is on the first day of treatment, instead of the third day in the study by Sutanto. The authors base this delay in dosing on a mathematical modeling study showing that postponing the dose increases its transmission blocking effect. An optimal effect is predicted with a single primaquine dose on day 8, related to the fact that in falciparum malaria the peak in gametocyte density is usually delayed by around 1–2 weeks after the initial acute attack [10]. However, the model depends on assumptions regarding the stage specificity of the gametocytocidal effect of primaquine, which might be broader than only the mature gametocytes, and the lack of a gametocytocidal effect of ACT. Postponing the primaquine dose allows additional time for transmission of the infection. A study from Myanmar on the gametocytocidal effect of a single dose of 0.45 mg/kg primaquine given on the first instead of the third day of treatment showed a beneficial effect on gametocyte clearance similar to that observed in the current study, as well as a similar favorable safety profile [11]. A directly observed primaquine dose on the first day of therapy might also be easier to implement operationally. A drawback is that the hemolytic effect of primaquine in patients with G6PD deficiency is increased in the presence of fever [5], which is higher at the start of treatment.

In conclusion, the study of Sutanto and colleagues clearly demonstrates the efficacy and safety of a single dose of 0.45 mg/kg primaquine on the third day of treatment as a gametocytocide drug in the treatment of uncomplicated falciparum malaria, excluding patients with G6PD deficiency. Dose and timing differ from the recently adopted WHO guideline, recommending a single dose of 0.25 mg/kg primaquine on the first day of treatment in similar settings, without prior testing for G6PD enzyme activity. Further studies will be needed to provide additional evidence for the optimal timing and dose of an efficacious yet safe deployment of primaquine as gametocytocidal and transmission-blocking treatment in populations with a high prevalence of G6PD deficiency.

## References

[1] Fairhurst RM, Nayyar GM, Breman JG (2012). Artemisinin-resistant malaria: research challenges, opportunities, and public health implications. Am J Trop Med Hyg.

[2] Maude RJ, Pontavornpinyo W, Saralamba S (2009). The last man standing is the most resistant: eliminating artemisinin-resistant malaria in Cambodia. Malar J.

[3] Sutanto I, Suprijanto S, Kosasih A (2013). The effect of primaquine on gametocyte development and clearance in the treatment of uncomplicated falciparum malaria with dihydroartemisinin-piperaquine in South Sumatra, Western Indonesia: an open-label, randomized controlled trial [published online ahead of print 21 November 2012]. Clin Infect Dis.

[4] Matsuoka H, Ishii A, Panjaitan W, Sudiranto R (1986). Malaria and glucose-6-phosphate dehydrogenase deficiency in North Sumatra, Indonesia. Southeast Asian J Trop Med Public Health.

[5] Cappellini MD, Fiorelli G (2008). Glucose-6-phosphate dehydrogenase deficiency. Lancet.

[6] World Health Organization Evidence Review Group The safety and effectiveness of single dose primaquine as a *P. falciparum* gametocytocide.

[7] Mackerras MJ, Ercole QN (1949). Observations on the action of quinine, atebrin and plasmoquine on the gametocytes of *Plasmodium falciparum*. Trans R Soc Trop Med Hyg.

[8] White NJ (2012). Primaquine to prevent transmission of falciparum malaria. Lancet Infect Dis.

[9] World Health Organization Single dose primaquine as a gametocytocide in *Plasmodium falciparum* malaria.

[10] Lawpoolsri S, Klein EY, Singhasivanon P (2009). Optimally timing primaquine treatment to reduce *Plasmodium falciparum* transmission in low endemicity Thai-Myanmar border populations. Malar J.

[11] Smithuis F, Kyaw MK, Phe O (2010). Effectiveness of five artemisinin combination regimens with or without primaquine in uncomplicated falciparum malaria: an open-label randomised trial. Lancet Infect Dis.

